# Effects of interdialytic interval on heart rate variability in chronic hemodialysis patients: a cross-sectional study

**DOI:** 10.1038/s41598-021-00093-0

**Published:** 2021-10-22

**Authors:** Kajohnsak Noppakun, Phasakorn Putchagarn, Arintaya Phrommintikul, Wanwarang Wongcharoen

**Affiliations:** 1 Division of Nephrology, Department of Internal Medicine, Faculty of Medicine, Chiang Mai University, Chiang Mai, Thailand; 2 Pharmacoepidemiology and Statistics Research Center (PESRC), Faculty of Pharmacy, Chiang Mai University, Chiang Mai, Thailand; 3 Division of Cardiology, Department of Internal Medicine, Faculty of Medicine, Chiang Mai University, Chiang Mai, 50200 Thailand

**Keywords:** Cardiology, Medical research, Nephrology

## Abstract

Previous studies showed that long interdialytic interval of chronic hemodialysis increased risk of sudden cardiac death compared to short interdialytic interval. Diabetes mellitus (DM) and autonomic dysfunction are the strong adverse predictors of survival in ESRD patients. We aimed to compare autonomic function between long and short interdialytic interval of chronic hemodialysis in patients with and without DM. One-hundred sixty-three patients receiving chronic hemodialysis were enrolled. The electrocardiogram recording was performed twice in each patient during 4-h hemodialysis session after long and short interdialytic intervals to assess heart rate variability (HRV). Mean age was 61.4 ± 14.3 years. HRV parameters during hemodialysis did not differ between long and short interdialytic interval in overall population. Nevertheless, in 82 (50.3%) patients, SDNN (47.4 ± 23.8 vs. 43.4 ± 19.5 ms, P = 0.039), ASDNN (24.8 ± 14.3 vs. 22.7 ± 12.3 ms, P = 0.025), LF (8.4 ± 6.8 vs. 7.6 ± 6.6 ms^2^, P = 0.040) increased after long interdialytic interval. The greater change of SDNN, ASDNN, VLF and LF between long and short interdialytic intervals was noted in DM, compared to non-DM patients. We demonstrated that there was no difference of HRV parameters after short and long interdialytic interval. However, there was greater autonomic alteration observed in DM than non-DM patients between 2 interdialytic intervals.

## Introduction

Cardiovascular diseases are the leading cause of death in patients with end-stage kidney disease (ESRD) requiring chronic dialysis. Previous studies have demonstrated that sudden cardiac death (SCD) accounts for approximately 25% of all-cause mortality and 70% of cardiovascular mortality in patients with ESRD^1–3^. Conventionally, hemodialysis therapy is scheduled thrice weekly, or even twice weekly in some patients who still have residual renal function^4^. Interestingly, the higher mortality and incidence of SCD has been reported to occur during the long interdialytic interval compared to the short interdialytic interval^5–7^. It has been described that numerous factors contribute to the heightened risk of SCD in chronic hemodialysis patients. Chronic hypervolemia in patients receiving hemodialysis may result in the structural and functional changes in myocardium, leading to the occurrence of arrhythmia. The oxidative stress, inflammation and abnormal calcium or phosphate metabolism, may accelerate the progression of atherosclerosis, leading to the increased risk of myocardial infarction^8,9^. In addition, electrolyte imbalance and the disorder in autonomic nervous system may result in the increased risk of SCD^10,11^.

Heart rate variability (HRV) has been used to assess the autonomic function in several conditions including patients with ESRD. Several investigators have demonstrated that HRV predicts long-term cardiovascular outcomes in patients receiving chronic hemodialysis^12–15^. Nevertheless, little is known regarding the effect of long and short interdialytic interval on HRV patients with chronic hemodialysis. As the long interdialytic interval is associated with increased risk of SCD, it is possible that HRV may differ between long and short interdialytic interval. The presence of diabetes mellitus (DM) is a strong predictor of adverse prognosis in ESRD patients. It is well-established that DM is associated with overall decrease in HRV^16^. However, one study showed that HRV could not predict the outcomes in ESRD with diabetes mellitus (DM)^14^.

Therefore, we aimed to examine the difference of HRV parameters between the long interdialytic interval and short interdialytic interval in chronic hemodialysis patients and to compare the change of HRV between DM and non-DM patients.

## Methods

### Study design and participants

We conducted a cross-sectional study to explore the relationship between HRV and dialytic interval with DM status as an effect modifier. We enrolled ESRD patients who had been receiving regular hemodialysis at hemodialysis unit, Chiang Mai University hospital, between April 2018 to December 2018. Patients who were older than 18 years and received regular hemodialysis for at least 3 months were eligible to enroll into the study. Patients were excluded if they had atrial fibrillation, atrial flutter or pacemaker installation. All subjects underwent Holter ECG monitoring for HRV assessment (GE Seer Light Extend, GE Medical Systems, Suzuken Company, Ltd.) The Holter ECG was monitored twice for 4 h during dialysis session on the day after short interdialytic interval and on the day after long interdialytic interval. All patients enrolled in the study had been receiving thrice weekly hemodialysis. The long interdialytic interval was 2-day interval between hemodialysis sessions and the short interdialytic interval was 1-day interval between hemodialysis sessions.

Clinical data were obtained from medical records including age, gender, co-morbidities, medications, duration of hemodialysis, biochemical analysis and ultrafiltration volume. Baseline DM status was determined by self-report, documented DM from medical record, and history of using or currently use of diabetic medications or insulin.

The study procedure was performed according to Declaration of Helsinki. Informed consent was obtained from all participants. This study was approved by the ethics committee of the Faculty of Medicine, Chiang Mai University, approval number 108/2561. It was registered in clinicaltrials.in.th, and the identification number is TCTR20180330002 (29 MAR 2018).

### HRV measurement

Before analyzing the data, the ECG recordings were manually preprocessed to exclude noise and other artifacts. Premature supraventricular and ventricular beats, missed beats, and pauses were filtered and replaced by an interpolated value.

Time-domain and frequency-domain analyses were performed according to the standard guidelines^17^. Time-domain HRV indexes were analyzed using statistical methods. The square root of the mean squared differences of successive normal-to-normal (NN) intervals (rMSSD), the standard deviation (SD) of all NN intervals (SDNN), the average of the SD of the 5-min NN intervals over the entire recording (ASDNN), the SD of the average NN intervals calculated over 5-min periods of the entire recording (SDANN), and the proportion of adjacent NN intervals differing by > 50 ms (pNN50) during the 10-min recording were measured. Frequency-domain HRV were analyzed using autoregressive power spectral analysis applied to the RR interval time series. The order of the autoregressive models used to estimate the power spectrum was calculated by commercially available software (MARS™ Ambulatory ECG System software version 8, developed by GE Healthcare).

The following spectral bands were identified: very low frequency (VLF) (0.003–0.04 Hz), low frequency (LF) (0.04–0.15 Hz) and high frequency (HF) (0.15–0.4 Hz). Total power (0–0.5 Hz) and the areas below each peak was calculated in absolute units (ms^2^). The normalization of LF (LF n.u.) and HF (HF n.u.) was also calculated in percentage. LF n.u. was defined as LF/ (total power-VLF) × 100. HF n.u. was defined as HF/ (total power-VLF) × 100. The absolute delta change of HRV parameters after long interdialytic interval and short interdialytic interval was defined as HRV parameters after long interdialytic interval—HRV parameters after short interdialytic interval.

### Statistical analysis

Results were expressed as mean ± SD, unless otherwise specified. The Kolmogorov–Smirnov test was used to assess normality of the data. The continuous variables were compared between groups with the paired or unpaired t test as appropriate. Mann–Whitney U test and Wilcoxon signed-rank test were used if data were not normally distributed. Categorical variables were summarized using frequencies and proportions and were compared using Chi-square or Fischer exact test as appropriate. P values < 0.05 were considered statistically significant. Statistical software package IBM SPSS Statistics for Windows, version 23.0 (IBM Corp., Armonk, NY, USA, https://www.ibm.com/products/spss-statistics) was used for analysis.

### Ethics approval and consent to participate

The study was registered in clinicaltrials.in.th, and the identification number is TCTR20180330002. The Effect of long and short interdialytic interval of chronic hemodialysis on heart rate variability in patients with ESRD was approved by the ethics committee of the Faculty of Medicine, Chiang Mai University, approval number 108/2561. The investigations were performed according to Declaration of Helsinki. Informed consent was obtained from all participants.

## Results

### Baseline characteristics

One-hundred sixty-three patients with ESRD receiving regular hemodialysis were enrolled in the study. Baseline clinical characteristics and biochemical data are shown in Table 1. The mean age of overall population was 61.4 ± 14.3 years. The mean left ventricular ejection fraction of 63.0 ± 12.3%. The median dialysis vintage was 3 (1–5) years. The primary cause of ESRD in nearly half of the patients (48.6%) was diabetic nephropathy. Other causes included glomerulonephritis (15.9%) and hypertensive nephropathy (15.2%). All patients had been receiving thrice weekly hemodialysis. Of 163 patients, 82 (50.3%) had DM and 81 (49.7%) did not have DM at baseline. Compared between DM and non-DM patients, DM patients are older (63.8 ± 10.4 vs. 58.9 ± 17.1, P = 0.025) and had higher prevalence of dyslipidemia. DM patients also had greater use of antiplatelet agents, statins and diuretics.

**Table 1:** Baseline characteristics.

	Total (N = 163)	DM (N = 82)	Non-DM (N = 81)	P value^a^
Age (years)	61.4 ± 14.3	63.8 ± 10.4	58.9 ± 17.1	0.025
Male	88 (54.0%)	57 (59.4%)	52 (50.0%)	0.203
Dialysis vintage^b^ (years)	3 (1–5)	3 (1–5)	4 (1–7)	0.057
LVEF (%)	63.0 ± 12.3	62.5 ± 13.1	63.5 ± 11.4	0.927
Kt/V	1.7 ± 0.3	1.6 ± 0.3	1.7 ± 0.3	0.314
Albumin (g/dL)	3.9 ± 0.4	4.0 ± 0.4	3.9 ± 0.5	0.149
Hemoglobin (g/dL)	10.5 ± 1.7	10.7 ± 1.6	10.3 ± 1.8	0.226
**Co-morbidities**				
Hypertension	151 (94.4%)	76 (93.8%)	75 (94.9%)	1.000
Dyslipidemia	111 (70.3%)	71 (88.8%)	40 (51.3%)	< 0.001
Coronary artery disease	24 (15.2%)	16 (20.0%)	8 (10.3%)	0.120
Cardiovascular disease	12 (7.6%)	6 (7.5%)	6 (7.7%)	1.000
Peripheral artery disease	4 (2.5%)	3 (3.8%)	1 (1.3%)	0.620
Chronic obstructive pulmonary disease	3 (1.9%)	1 (1.3%)	2 (2.6%)	0.618
**Medications**				
ACEI/ARB	56 (35.7%)	26 (32.9%)	30 (38.5%)	0.508
Beta-blockers	104 (66.2%)	53 (67.1%)	51 (65.4%)	0.867
Calcium channel blockers	117 (74.5%)	56 (70.9%)	61 (78.2%)	0.360
Diuretics	75 (47.8%)	52 (65.8%)	23 (29.5%)	< 0.001
Alpha blockers	52 (33.1%)	28 (35.4%)	24 (30.8%)	0.612
Statins	107 (68.2%)	65 (82.3%)	42 (53.8%)	< 0.001
Anti-platelets	60 (38.2%)	48 (60.8%)	12 (15.4%)	< 0.001
Oral anticoagulants	11 (7.0%)	6 (7.6%)	5 (6.4%)	1.000

*ACEI/ARB* angiotensin converting enzyme inhibitor/angiotensin receptor blocker, *DM *diabetes mellitus, *LVEF* left ventricular ejection fraction.

^a^P value from the comparisons between DM versus non-DM using unpaired t test or Chi-square test unless otherwise specified.

^b^Median (interquartile range) and were compared between groups using Mann–Whitney U test.

### Clinical and biochemical data between short and long interdialytic intervals

The interdialytic weigh gain was greater after long interdialytic interval than that after short interdialytic interval (2.2 ± 1.0 vs. 1.7 ± 1.0 kg, P < 0.001). The fluid removal volume was also greater after long interdialytic interval than that after short interdialytic interval (2,411 ± 1,015 vs. 2,005 ± 950 mL, P < 0.001). There was no difference of blood pressure prior hemodialysis when measured on the day after long interdialytic interval and after short interdialytic interval. We did not observe the increase in serum potassium after long interdialytic interval compared to that after short interdialytic interval. On the contrary, we found that serum potassium was lower after long interdialytic interval compared to short interdialytic interval in DM patients (4.3 ± 0.6 vs. 4.4 ± 0.6, P = 0.03). No difference of serum potassium was noted after long and short interdialytic intervals in non-DM patients. (Table 2).

**Table 2:** The clinical and biochemical data between short and long interdialytic intervals in patients with and without diabetes mellitus.

	Total (N = 163)		P value^a^	DM (N = 82)		P value^a^	Non-DM (N = 81)		P value^a^
	Short interdialytic interval	Long interdialytic interval		Short interdialytic interval	Long interdialytic interval		Short interdialytic interval	Long interdialytic interval	
Interdialytic weight gain (kg)	1.7 ± 1.0	2.2 ± 1.0	< 0.001	1.8 ± 1.0	2.2 ± 1.0	< 0.001	1.7 ± 1.0	2.0 ± 1.0	< 0.001
Net ultrafiltration (mL)	2005 ± 950	2411 ± 1015	< 0.001	2097 ± 1000	2529 ± 1068	< 0.001	1911 ± 893	2292 ± 950	< 0.001
Pre-dialysis SBP (mmHg)	143 ± 20	146 ± 20	0.083	145 ± 22	147 ± 21	0.273	141 ± 19	144 ± 19	0.181
Pre-dialysis DBP (mmHg)	74 ± 13	75 ± 13	0.685	71 ± 12	72 ± 13	0.689	77 ± 13	78 ± 14	0.856
Post-dialysis SBP (mmHg)	143 ± 18	145 ± 18	0.201	140 ± 17	145 ± 19	0.022	146 ± 19	145 ± 17	0.689
Post-dialysis DBP (mmHg)	77 ± 12	77 ± 12	0.936	72 ± 10	73 ± 11	0.114	82 ± 11	80 ± 12	0.256
Pre-dialysis serum sodium (mmol/L)	137 ± 3	137 ± 3	0.077	137 ± 3	136 ± 3	0.126	138 ± 3	138 ± 3	0.337
Pre-dialysis serum potassium (mmol/L)	4.4 ± 0.6	4.3 ± 0.6	0.125	4.4 ± 0.6	4.3 ± 0.6	0.029	4.4 ± 0.6	4.4 ± 0.7	0.686

*DBP* diastolic blood pressure, *DM* diabetes mellitus, *SBP* systolic blood pressure.

^a^P value from the comparisons between short versus long interdialytic interval using paired t test.

### The HRV after long interdialytic interval and short interdialytic interval

Table 3 shows the comparison of HRV during 4-h hemodialysis between short and long interdialytic interval in overall population. We demonstrated that all HRV parameters did not differ between short and long interdialytic interval.

**Table 3:** Heart rate variability during 4-h hemodialysis between short and long interdialytic interval in overall population.

HRV parameters	Total (N = 163)		P value*
	Short interdialytic interval	Long interdialytic interval	
Mean NN (ms)	869.8 ± 136.0	864.8 ± 132.8	0.369
SDNN (ms)	49.1 ± 21.5	49.3 ± 23.7	0.946
SDANN (ms)	40.0 ± 18.1	38.6 ± 19.9	0.714
rMSSD (ms)	21.2 ± 16.8	22.3 ± 24.1	0.805
pNN50 (%)	4.6 ± 10.1	4.2 ± 8.5	0.952
VLF (ms^2^)	15.4 ± 8.3	15.6 ± 9.0	0.916
LF (ms^2^)	9.9 ± 7.8	10.0 ± 8.0	0.756
LF normalized unit (n.u.)	51.9 ± 10.8	52.5 ± 10.8	0.381
HF (ms^2^)	8.6 ± 6.5	8.5 ± 6.1	0.960
HF normalized unit (n.u.)	48.1 ± 10.8	47.5 ± 10.8	0.381
LF/HF ratio	1.2 ± 0.5	1.2 ± 0.5	0.681

*P < 0.05 from the comparison between short interdialytic interval and long interdialytic interval using unpaired.

The change of HRV parameters after long and short interdialytic intervals were analyzed according to diabetic status. (Table 4) In 81 non-DM patients, there were no differences of time-domain and frequency-domain HRV parameters between long and short interdialytic intervals. However, in 82 (50.3%) DM patients, SDNN (47.4 ± 23.8 ms vs. 43.4 ± 19.5 ms, P = 0.039), ASDNN (24.8 ± 14.3 ms vs. 22.7 ± 12.3 ms, P = 0.025), LF (8.4 ± 6.8 ms^2^ vs. 7.6 ± 6.6 ms^2^, P = 0.040) increased after long interdialytic interval. Nevertheless, there was no difference in LF n.u., HF n.u. and LF/HF ratio between 2 intervals.

**Table 4:** Heart rate variability between short and long interdialytic interval, according to diabetic status.

HRV parameters	DM patients (N = 82)		P value	Non-DM patients (N = 81)		P value
	Short interdialytic interval	Long interdialytic interval		Short interdialytic interval	Long interdialytic interval	
Mean NN (ms)	862 ± 115	852 ± 116	0.113	878 ± 155	878 ± 147	0.731
SDNN (ms)	43.4 ± 19.5	47.4 ± 23.8	0.039*	54.8 ± 22.0	51.3 ± 23.6	0.059
SDANN (ms)	34.9 ± 16.7	38.5 ± 21.4	0.125	41.0 ± 19.0	38.7 ± 18.4	0.291
ASDNN (ms)	22.7 ± 12.3	24.8 ± 14.3	0.025*	39.2 ± 59.3	31.2 ± 15.7	0.051
rMSSD (ms)	18.3 ± 14.6	22.4 ± 31.3	0.338	24.2 ± 18.3	22.1 ± 13.6	0.238
pNN50 (%)	3.5 ± 8.9	4.3 ± 9.6	0.239	5.4 ± 11.1	4.2 ± 7.3	0.280
VLF (ms^2^)	12.5 ± 7.2	13.5 ± 8.5	0.153	18.4 ± 8.4	17.7 ± 9.0	0.174
LF (ms^2^)	7.6 ± 6.6	8.4 ± 6.8	0.040*	12.3 ± 8.2	11.7 ± 8.8	0.210
LF normalized unit (n.u.)	49.9 ± 10.0	51.1 ± 11.9	0.297	53.8 ± 10.3	54.0 ± 9.3	0.968
HF (ms^2^)	7.2 ± 5.7	7.5 ± 5.8	0.299	10.1 ± 6.8	9.5 ± 6.2	0.398
HF normalized unit (n.u.)	50.0 ± 11.0	48.9 ± 11.9	0.297	46.1 ± 10.3	46.0 ± 9.3	0.968
LF/HF ratio	1.1 ± 0.5	1.1 ± 0.5	0.486	1.3 ± 0.4	1.3 ± 0.5	0.908

*P value from the comparison between short versus long interdialytic interval using paired t test.

The absolute delta change of HRV parameters after long and short interdialytic intervals was compared between DM and non-DM patients. (Table 5) We demonstrated the greater increment of SDNN (+ 4.0 ± 17.1 ms vs. −3.5 ± 20.2 ms, P = 0.006), ASDNN (+ 2.1 ± 8.1 ms, vs. −8.0 ± 54.9 ms, p = 0.003), VLF (+ 1.0 ± 5.1 ms^2^ vs. −0.7 ± 7.7 ms^2^, P = 0.040) and LF (+ 0.7 ± 4.6 ms^2^vs. −0.6 ± 9.0 ms^2^, P = 0.040) after long interdialytic interval compared to short interdialytic interval in DM than non-DM patients.

**Table 5:** The change of HRV parameters between long and short interdialytic intervals, compared between DM and non-DM patients.

∆ HRV parameters	DM patients (N = 82)	Non-DM patients (N = 81)	P value*
∆ SDNN (ms)	+ 4.0 ± 17.1	−3.5 ± 20.2	0.006
∆ SDANN (ms)	+ 3.6 ± 18.3	−2.3 ± 18.3	0.075
∆ ASDNN (ms)	+ 2.1 ± 8.2	−8.0 ± 54.9	0.003
∆ rMSSD (ms)	+ 4.2 ± 28.9	−2.1 ± 14.7	0.125
∆ pNN50 (%)	+ 0.8 ± 7.1	−1.2 ± 10.5	0.100
∆ VLF (ms^2^)	+ 1.0 ± 5.1	−0.7 ± 7.7	0.040
∆ LF (ms^2^)	+ 0.7 ± 4.6	−0.6 ± 9.0	0.040
∆ LF normalized unit (n.u.)	+ 1.1 ± 7.8	+ 0.1 ± 8.6	0.418
∆ HF (ms^2^)	+ 0.3 ± 3.9	−0.6 ± 6.0	0.189
∆ HF normalized unit (n.u.)	−1.1 ± 7.8	−0.1 ± 8.6	0.418
∆ LF/HF ratio	0 ± 0.4	0 ± 0.4	0.582

∆ = absolute change of HRV parameters between long and short interdialytic intervals, *DM*  diabetes mellitus, *HRV* heart rate variability.

*P value from the comparison between DM and non-DM patients using unpaired t test.

## Discussion

Sudden cardiac death has become a great concern in the management of patients with ESRD receiving chronic hemodialysis^1,2^. It has been described that autonomic dysfunction plays an important role in the occurrence of SCD^18^. Previous study has demonstrated the sympathetic overactivity and vagal withdrawal in patients with ESRD receiving chronic hemodialysis. Furthermore, the investigators found that the autonomic dysfunction was associated with higher left ventricular mass and poorer physical performance in chronic hemodialysis patients^19^.

Several observational studies have shown that the incidence of SCD significantly increased during the period after long interdialytic interval compared to that after short interdialytic interval^5,6^. The significant alteration of fluid and electrolytes fluxes may account for the worse outcomes after long interdialytic interval. In addition, the acid–base imbalance and the change in left ventricular mechanics may contribute to the heightened risk after long interdialytic interval. It is possible that these alterations after long interdialytic interval may result in the change of autonomic function, leading to the increased risk of SCD during the period after long interdialytic interval. Several investigators have reported that HRV predicts long-term outcomes in ESRD patients receiving hemodialysis^12–14^.

In this study, we observed that there was no difference of HRV parameters after short and long interdialytic interval in overall population. With this regard, the contribution of autonomic dysfunction to the increased risk of SCD after long interdialytic interval may be relatively low, compared to other strong risk factors^20^.

The autonomic dysfunction is highly prevalent in DM patients^16^. The decrease in overall HRV has been described in patients with DM and is associated with poor prognosis^21^. Therefore, we also analyzed HRV parameters according to diabetic status. We demonstrated that HRV parameters were comparable between the periods after long and short interdialytic intervals in non-DM patients. Nevertheless, in DM patients, the greater increment in SDNN, ASDNN, VLF and LF was evident after long interdialytic interval than after short interdialytic interval. It is plausible that the autonomic dysfunction in DM patients may account for the greater difference of HRV parameters between long and short interdialytic interval than non-DM patients^22^. Previous studies suggest that VLF is influenced by the renin–angiotensin system and is also associated with the sympathetic activity^23–26^. LF reflects both sympathetic activity and vagal activity. Several investigators have described that the increase in LF may indicate the increased sympathetic activity^23,27^. As a result, the activity of renin–angiotensin system and sympathetic system may increase after long interdialytic interval compared to short interdialytic interval in DM patients. Whether these changes in HRV parameters can explain the increased risk of SCD after long interdialytic interval in DM patients merits further study.

This study has some limitations. It is a single center study although the number of participants in the study is relatively large. Regarding the generalizability of our study, the studied participants had baseline characteristics as well as concomitant medications including beta-blockers which were comparable to other studies^7,28^. Therefore, we expect that our results can be applied to other populations. Lastly, we did not adjust P-values for multiple statistical testing in our study as this measure may increase the chance of making a type II error^29^. The quality and an effect size of our study should be considered in accordance with an interpretation of statistical significance.

## Conclusion

We demonstrated that there was no difference of HRV parameters after short and long interdialytic interval. According to diabetic status, there was greater autonomic alteration observed in DM patients between short and long interdialytic intervals than in non-DM patients.

## Data Availability

The informed consent given by effect of long and short interdialytic interval of chronic hemodialysis on heart rate variability in patients with ESRD study participants does not cover data posting in public databases. However, data are available upon request should be sent to bwanwarang@yahoo.com and are subject to approval by the Faculty of Medicine, Chiang Mai University Ethics Committee.
